# *In Vivo* Tumor Targeting and Imaging with Engineered Trivalent Antibody Fragments Containing Collagen-Derived Sequences

**DOI:** 10.1371/journal.pone.0005381

**Published:** 2009-04-29

**Authors:** Ángel M. Cuesta, David Sánchez-Martín, Laura Sanz, Jaume Bonet, Marta Compte, Leonor Kremer, Francisco J. Blanco, Baldomero Oliva, Luis Álvarez-Vallina

**Affiliations:** 1 Molecular Immunology Unit, Hospital Universitario Puerta de Hierro, Madrid, Spain; 2 Structural Bioinformatics' Lab, Biomedical Informatics Research Unit, Parc de Recerca Biomèdica de Barcelona, Barcelona, Spain; 3 Protein Tools Unit, Centro Nacional de Biotecnología, Consejo Superior de Investigaciones Científicas, Madrid, Spain; 4 Structural Biology Unit, CIC bioGUNE, Parque Tecnológico de Bizkaia, Bizkaia, Spain; Tufts University, United States of America

## Abstract

There is an urgent need to develop new and effective agents for cancer targeting. In this work, a multivalent antibody is characterized *in vivo* in living animals. The antibody, termed “trimerbody”, comprises a single-chain antibody (scFv) fragment connected to the N-terminal trimerization subdomain of collagen XVIII NC1 by a flexible linker. As indicated by computer graphic modeling, the trimerbody has a tripod-shaped structure with three highly flexible scFv heads radially outward oriented. Trimerbodies are trimeric in solution and exhibited multivalent binding, which provides them with at least a 100-fold increase in functional affinity than the monovalent scFv. Our results also demonstrate the feasibility of producing functional bispecific trimerbodies, which concurrently bind two different ligands. A trimerbody specific for the carcinoembryonic antigen (CEA), a classic tumor-associated antigen, showed efficient tumor targeting after systemic administration in mice bearing CEA-positive tumors. Importantly, a trimerbody that recognizes an angiogenesis-associated laminin epitope, showed excellent tumor localization in several cancer types, including fibrosarcomas and carcinomas. These results illustrate the potential of this new antibody format for imaging and therapeutic applications, and suggest that some laminin epitopes might be universal targets for cancer targeting.

## Introduction

An optimized antibody fragment designed for targeting cancer *in vivo* should fulfill several requirements: high specificity and affinity for the target antigen, low immunogenicity; and be ready available form expression to purified protein [1]. The pharmacokinetic properties of the antibody should be adjusted depending on the intended use. Format and molecular weight of tumor targeting antibodies are critical factors that influence their pharmacokinetics. Intact IgG molecules (150 kDa) display low blood clearance and incomplete tumor penetration. On the other hand, small monovalent single-chain variable fragments (scFv) (25–30 kDa) are more effective in tumor penetration but they are cleared too rapidly and have poor tumor retention because of their binding properties [2].

The ideal tumor-targeting antibodies are intermediate-sized multivalent molecules, which provide rapid tissue penetration, high target retention and rapid blood clearance. Recent biodistribution studies [3] indicate that bivalent antibodies such as diabodies (60 kDa), and minibodies (80 kDa) may be best suited for tumor imaging and therapy due to a higher total tumor uptake and better tumor-to-blood ratios than intact IgG molecules. Diabodies are non-covalent dimeric molecules spontaneously formed in scFv with short linkers connecting the variable region genes [4], [5]. Another useful format derived from scFv, with expanded half-life but still rapid, high-level uptake into tumors is the minibody, which results from the fusion of scFv with the IgG1 CH3 domain, which provokes dimerization [6].

However, despite of the good results obtained with these engineered formats in various models [3], [7]–[12], there are still some limitations that need to be dealt with in order to take full advantage of the targeting capability of these recombinant antibodies. One of these drawbacks is their relatively limited flexibility, and the necessity of the second antigen to be precisely oriented and located in a strictly defined area once the antibody binds the first antigen [13], [14]. Therefore, bound antigens should be almost opposed in the diabody, and in a small circular area in the minibody, which actually precludes the binding to the second antigen in a number of situations. This implies that part of the increased affinity observed relies mainly on binding/rebinding, and not on simultaneous binding to different molecules of the antigen. To circumvent these drawbacks we have developed a new class of multivalent antibodies. These antibodies, termed “trimerbodies”, use the N-terminal association subdomain of collagen XVIII NC1, responsible for the non-covalent trimerization of collagen alpha chains, to drive multimerization [15].

Until now, most of the tumor targeting agents have focused on tumor-associated cell surface markers, such as the carcinoembryonic antigen (CEA). The CEA is a heavily glycosylated cell adhesion molecule that is widely used as marker for colorectal, stomach, pancreas, breast, and lung carcinomas; and several other carcinomas of epithelial origin [16]. However, molecules, which are selectively expressed in the stroma and in angiogenesis-active sites, appear to be particularly suited for antibody-based strategies for targeting solid tumors. During tumor progression, the extracellular matrix suffers extensive remodeling through deposition of new components and proteolytic degradation, giving rise to unique epitopes not usually accessible in homeostatic organs [17].

In the present study, we characterized the binding affinity *in vitro* and the *in vivo* tumor targeting properties of trimerbodies with specificity for human CEA, and an angiogenesis-associated laminin epitope. A trimerbody with specificity for the hapten NIP (4-hydroxy-5-iodo-3-nitrophenyl) was used as control. All the purified trimerbodies exhibited excellent antigen binding capacity and were multivalent, which provides them with a significant increase in functional affinity. Fluorescently labeled anti-CEA trimerbodies showed efficient tumor targeting of colorectal carcinomas in mice, and importantly, anti-laminin trimerbodies showed excellent tumor localization in several cancer types, including fibrosarcomas and carcinomas. These results illustrate the potential of this novel antibody format for imaging and therapeutic applications.

## Materials and Methods

### Antibodies and Reactives

The monoclonal antibodies (mAbs) used included 9E10 (Abcam, Cambridge, UK) specific for human c-myc, and NCRC23 (AbD Serotec, Kidlington, UK) specific for human CEA. The polyclonal antibodies, a rabbit anti-bovine serum albumin (BSA), a horseradish peroxidase (HRP)-conjugated goat anti-rabbit IgG, and an HRP-conjugated goat anti-mouse IgG (Fc specific) were from Sigma-Aldrich (St. Louis, MO, USA). Laminin extracted from the Engelbreth-Holm-Swarm (EHS) mouse tumor was from Becton Dickinson Labware (Bedford, MA, USA). Human CEA and BSA were from Sigma-Aldrich. BSA was conjugated with 4-hydroxy-5-iodo-3-nitrophenyl (NIP) (Sigma-Aldrich) in a molar ratio of 10∶ 1 (NIP_10_-BSA) as described [18].

### Cells and culture conditions

HEK-293 cells (human embryo kidney epithelia; CRL-1573), and its derivative 293T cells (CRL-11268), HT-1080 (human fibrosarcoma; CCL-121), MKN45 cells (human stomach adenocarcinoma; JCRB-0254), and HeLa cells (human cervix carcinoma; CCL-2) were cultured in Dulbecco's modified Eagle's medium (DMEM) supplemented with 10% (vol/vol) heat-inactivated Fetal Calf Serum (FCS) (all from Invitrogen, Carlsbad, CA) referred as to DMEM complete medium (DCM). HeLa^CEA^ cells [19] were grown in DCM supplemented with 750 µg/ml G418 (Invitrogen).

### Construction of expression vectors

pCR3.1-L36 and pCR3.1-L36-NC1^ES-^ expression vectors were constructed as described [17], [20]. The plasmid pVOM1.C23 containing the MFE-23 (anti-human CEA) scFv gene was kindly provided by Dr. R. E. Hawkins (University of Manchester, UK). The MFE-23 expression cassette was subcloned as HindIII-NotI into the vector pCEP4.6xHis-myc [20], resulting in pCEP4-MFE-23. Plasmid pCEP4-B1.8 containing the B1.8 (anti-hapten NIP) scFv gene and the polyhistidine and c-myc epitopes was constructed as described [20]. To construct the plasmids pCEP4-MFE-23-NC1^ES-^ and pCEP4-B1.8-NC1^ES-^ the 252 bp NotI fragment derived from the plasmid pCR3.1-L36-NC1^ES-^ was ligated into the NotI digested backbone of plasmids pCEP4-MFE-23 or pCEP4-B1.8 respectively.

### Cell transfections and purification of recombinant antibodies

HEK-293 or 293T cells were transfected with the appropriate expression vectors, using Superfect (QIAGEN GmbH, Hilden, Germany). To generate stable cell lines, pCR3.1-L36, and pCR3.1-L36-NC1^ES-^ transfected HEK-293 cells were selected in DCM with 500 µg/ml G418; pCEP4-MFE-23-NC1^ES-^ and pCEP4-B1.8-NC1^ES-^ transfected HEK-293 cells were selected in DCM with 150 µg/ml hygromycin B (Invitrogen). Supernatants from transient and stable transfected cell populations were analyzed for protein expression by ELISA, SDS-PAGE and Western blotting using anti-myc mAb. Stable transfected HEK-293 cells were used to collect serum-free conditioned media medium (∼1 liter) was concentrated (×10) with a 10.000 MWCO Vivaflow 50 filter (Vivascience AG, Hannover, Germany), dialyzed against PBS (pH 7.4) and loaded onto a HisTrap HP 1 ml column using an ÄKTA Prime plus system (GE Healthcare, Uppsala, Sweden). The purified antibodies were dialyzed against PBS, analyzed by SDS-PAGE under nonreducing or reducing conditions, and stored at −20°C.

### Analytical gel filtration chromatography

Experiments were performed at room temperature with an ÄKTA FPLC system (GE Healthcare) using a Superdex 200 10/300GL column in PBS. Samples of 100 µl at a concentration of 0.5–1.0 mg/ml were injected and eluted at a flow rate of 0.5 ml/min. The column was calibrated with blue dextran (excluded volume) and molecular weight markers from 16 to 655 kDa (GE Healthcare).

### ELISA

The ability of purified trimerbodies (L36, MFE23, or B1.8) to bind murine laminin, human CEA, or NIP_10_-BSA conjugates was studied by ELISA as described [21]. The multivalence of trimerbody molecules was studied by ELISA using supernatant from single (pCR3.1-L36-NC1^ES-^ or pCEP4-B1.8-NC1^ES-^) and double (pCR3.1-L36-NC1^ES-^ and pCEP4-B1.8-NC1^ES-^) transfected 293T cells. Maxisorp (NUNC Brand Products, Roskilde, Denmark) plates were coated with laminin (0.5 µg/well), and after washing and blocking with 200 µl 5% non-fat dry milk in PBS, 100 µl of supernatant from single or double transfected 293T cells were added for 1 hour at room temperature. After three washes, 100 µl of NIP_10_-BSA (10 µg/ml) were added for 1 hour at room temperature. After three washes, 100 µl of rabbit anti-BSA antibody (1∶1000) in 0.05% Tween-20-PBS were added for 1 hour at room temperature. After three more washes 100 µl of HRP-conjugated goat anti-rabbit IgG were added for 1 hour at room temperature, after which the plate was washed and developed.

### Flow Cytometry

The expression of CEA on HeLa, and HeLa^CEA^ cells and the binding of recombinant antibodies was studied as described [22]. Briefly, cells were incubated with anti-CEA mAb (5 µg/ml) or purified trimerbodies (anti-NIP or anti-CEA, 10 µg/ml) and mAb 9E10 (4 µg/ml) in 100 µl for 45 min. After washing, the cells were treated with appropriate dilutions of FITC-conjugated goat anti-mouse IgG (Sigma-Aldrich). The samples were analyzed with an EPICS XL (Coulter Electronics, Hialeah, FL, USA).

### Surface plasmon resonance analysis

Analyses were performed at room temperature using a BIAcore 3000 (GE Healthcare). CM5 dextran sensor chips (GE Healthcare) were used in all analyses, with HBS-EP running buffer (0.01 M HEPES pH 7.4, 0.15 M NaCl, 3 mM EDTA, 0.005% Surfactant P20), which was filtered with a 0.22 µm filter and degassed before use. Proteins were dissolved in 10 mM sodium acetate, pH 4.5. NIP_10_-BSA was directly immobilized on the sensor chip surface as recommended in the BIAapplications Handbook (GE Healthcare), in independent flow cells at approximately 100, 1800 and 8500 resonance units (RU). BSA was immobilized at 1900 on reference flow cell. After each experiment, surfaces were regenerated with 30 mM HCl, allowing resonance signals to return to baseline values. Analyses were performed in duplicate.

For kinetic analyses, a flow cell with small amounts of immobilized NIP_10_-BSA (∼100 RU) was used to minimize mass transport effects and rebinding. Individual samples consisting of purified scFv or trimerbody were passed over the chip surface at a flow rate of 20 µl/min and association/dissociation was measured. Bulk refractive index changes were removed by subtracting the reference flow cell responses; the average response of a blank injection was also subtracted from all sensorgrams. Data were analyzed using BIAevaluation v4.1 software provided with the biosensor, and kinetic data were fitted to the Langmuir 1∶1 interaction model.

### Molecular Modeling

The structure of the binding domain (L36 scFv) was modeled by comparative modeling [23] using as template the structure of 2GHW.B from the Protein Data Bank (PDB) [24]. This template was obtained with blast [25] e-value 2e-79 and a 70% of identity. The structure of the N-terminal trimerization domain of murine collagen XVIII NC1 was obtained from ModBase [26]. Both domains were linked by a 21 amino acid long peptide forming the L36 trimerbody monomer. The coordinates of the other two monomers were obtained applying a three-fold rotation axis symmetry. The trimer was formed by merging the coordinates of the three monomers. The structure of the trimer was optimized with GROMACS [27] and its energy was evaluated with DFIRE [28]. In order to compare the values of DFIRE between the monomer and the trimer, the energies were normalized dividing them by the length of the sequence.

### Serum stability

To determine whether trimerbody constructs remained functional in serum, five hundred nanograms of purified L36 trimerbody were incubated in 12.5% mouse serum from BALB/c mice (Harlan Ibérica, Barcelona, Spain) at 37°C for up to 72 h. Samples were removed for analysis at 3 h, 24 h, and 72 h following the start of incubation and frozen until the entire study was completed. As a control, a second set of serum-exposed samples was frozen immediately to represent a zero time point. Trimerbodies were then tested for their capability to retain functional binding to murine laminin by ELISA.

### Antibody labeling with cyanine 5

Purified L36 scFv and trimerbodies were labeled with cyanine 5 (Cy5) NHS esters (GE Healthcare) according to the manufacturer's recommendations. Cy5-labeled recombinant antibodies were separated from unincorporated Cy5 dye by gel-filtration on Sephadex G25-M (PD-10 Columns, GE Healthcare), and concentrated in 10.000 MWCO Vivaspin 500 filter (Vivascience) to approximately 1 mg/ml. The labeling ratio of Cy5 to antibody (Cy5∶antibody) was calculated as described [29] and was close to 1∶1. The functionality of Cy5-labeled antibodies was verified by ELISA against specific antigen.

### Infrared immunophotodetection in tumor-bearing mice

Wild-type MKN45, HT1080, or HeLa cells (1–2×10^6^) were implanted subcutaneously (n = 4) into the dorsal space of 6-week-old female Hsd: Athymic Nude-*Foxn1^nu^* mice (Harlan Ibérica). Nodule dimensions were used to calculate tumor volume using the formula: width^2^×length×0.52. When tumors reached an appropriate volume (0.2–0.4 cm^3^), mice were injected in the tail vein with 100 µl Cy5-labeled antibody solution in PBS (5 mg/Kg). Mice were imaged using the high-resolution charge-coupled-device (CCD) cooled digital camera ORCA-2BT (Hamamatsu Photonics France, Massy, France), and Wasabi software (Hamamatsu Photonics), under anesthesia. All mice were handled in accordance with the guidelines of the Hospital Universitario Puerta de Hierro Animal Care and Use Committee and performed in accordance with Spanish legislation.

## Results

### Design and Expression of Trimerbody Constructs

Structural analysis of the NC1 domain of collagen XVIII suggests that it consists of three segments, an N-terminal trimerization domain implicated in self-assembly of homotrimers; a central protease-sensitive hinge region; and the compact C-terminal endostatin domain [30]. We have previously shown that an engineered antibody containing the anti-laminin L36 scFv and the N-terminal trimerization domain of murine collagen XVIII NC1 was produced in human 293 cells in a functional active form [15]. To provide sufficient spatial flexibility to the N-terminal scFv a twenty-one amino acid artificial linker was used ([15], and Fig. S1).

**Figure 1:**
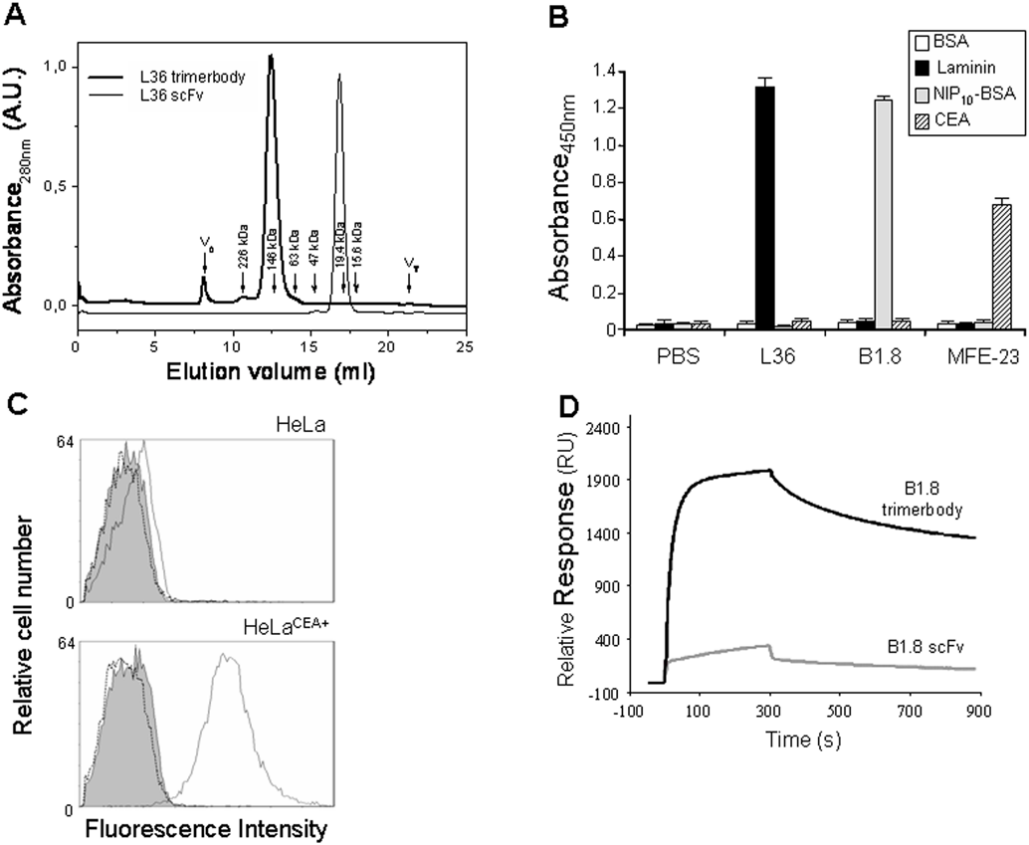
Molecular characterization of purified trimerbodies. (A) Elution profile of the gel filtration experiment of L36 constructs (scFv and scFv-NC1 “trimerbody”). The exclusion (V_e_) and total (V_T_) volumes are indicated. The elution volumes of selected molecular weight markers are also indicated with arrows and the corresponding molecular weights. For the shake of clarity the absorbance of both chromatograms have been scaled and shifted in the figure. The functionality of purified trimerbodies was demonstrated by ELISA (B) against plastic immobilized BSA, murine EHS laminin, NIP-BSA conjugates (NIP_10_-BSA), and human CEA; and by flow cytometry analysis (C) on CEA-negative and CEA-positive tumor cells. Isotype control (filled histogram), anti-CEA trimerbody (solid line), and anti-NIP trimerbody (dotted line) are shown. (D) Analysis of B1.8 scFv and B1.8 trimerbody binding to NIP_10_-BSA using BIAcore. The curves showed data obtained after subtraction of the binding response to a BSA-coated reference surface (1900 RU), to remove the effects of non-specific binding. Representative sensorgrams corresponding to the adjusted binding curves of affinity-purified B1.8 scFv and B1.8 trimerbody (dil. 1200, and 800 nM) in buffer HBS-EP injected over the same two flow cells as above. Antibody samples and running buffer were injected over the surfaces (5 min, flow rate 20 µl/min) and the dissociation phase was monitored for 8 min.

The trimeric nature of the purified scFv-NC1 fusion was demonstrated by ultracentrifugation [15] and analytical gel filtration chromatography (Fig. 1A). The elution of L36 scFv in an analytical gel filtration shows that it is monomeric. The calibration of the column using the markers yields a molecular weight of 24.2 kDa, in good agreement with the theoretical one (26.9 kDa). By contrast, the elution of L36 scFv-NC1 fusion corresponds to an estimated MW of 109.3 kDa, indicating that it is a trimer (the theoretical mass of the trimer is 111.4 kDa), in agreement with the previously reported analysis by analytical ultracentrifugation [15]. This trimeric antibody format was designated “trimerbody”.

In this study, we have extended the concept by designing trimerbodies with specificity for the hapten NIP or CEA. The scFv genes derived from the anti-NIP B1.8 [18] and the anti-CEA MFE-23 [31] antibodies were similarly assembled and expressed as soluble secreted proteins in human HEK293 cells and purified from conditioned medium by immobilized metal affinity chromatography. The purification scheme yielded antibodies that were >95% pure by SDS–PAGE (data not shown). Both B1.8 and MFE-23 trimerbodies eluted from the column as single peaks (data not shown) comparable to that shown in Figure 1A.

### Antigen Binding Activity

The functionality of purified trimerbodies was demonstrated by ELISA against plastic immobilized NIP-BSA conjugates (NIP_10_-BSA), murine EHS laminin, and human CEA (Fig. 1B). Their ability to detect the antigen in a cellular context was investigated by immunofluorescence labeling of human tumor cells that express CEA on the cell surface. Fluorescence staining was observed after incubation with MFE-23 trimerbody, followed by reaction with anti-myc mAb and detection with FITC-conjugated goat anti-mouse antibodies. In contrast, incubation of CEA-positive cells with B1.8 trimerbody or incubation of CEA-negative HeLa cells with MFE-23 trimerbody revealed no staining (Fig. 1C). These results demonstrated that trimerbodies recognized not only immobilized antigen, but also native antigen on the surface of tumor cells.

Surface plasmon resonance (SPR) was used to determine the influence of the scFv and trimerbody formats on B1.8 antibody function. We compared the binding curves for each antibody format using three different densities of NIP_10_-BSA covalently bound to the chip surface. Dextran-coupled BSA was used in a reference flow cell. To compare binding responses during association and dissociation, several concentrations of purified B1.8 antibodies were injected, ranging from 9 to 1,200 nM for B1.8 scFv and from 6 to 800 nM for B1.8 trimerbody onto the NIP_10_-BSA (1900 RU) surface (data not shown). In these conditions, only the trimerbody approached saturation of the antigen surface, whereas the scFv bound slowly and showed more rapid apparent dissociation. Sensorgrams indicated that the trimeric antibody has greater binding capacity than its monovalent counterpart (Fig. 1D).

For kinetic analyses, a flow cell coated with ∼100 RU of NIP_10_-BSA was used. In these assays, the B1.8 scFv had a profile considerably different from that of the B1.8 trimerbody, with slower association and faster dissociation rates. The kinetic rate constants for association (k_a_) and dissociation (k_d_) were determined by simultaneous fitting (global fitting) using the Langmuir 1∶1 interaction model. For B1.8 scFv, the apparent association and dissociation rate constants were k_a_ 1.0×10^4^±1.7×10^2^ M^−1^ s^−1^ and k_d_ 2×10^−3^±4×10^−5^ s^−1^ (Chi_2_ = 0.189). Using K_D_ = k_d_/k_a_, the equilibrium dissociation constant for B1.8 scFv was estimated as 2×10^−7^ M. Depending on the antibody concentration tested in each cycle, the trimerbody injected onto the low surface density of NIP_10_-BSA could probably bind both mono- and multivalently, and the apparent kinetic binding constants might include avidity effects of multivalent binding. Using a 1∶1 interaction model in which association and dissociation phases were treated separately for each concentration, we also fitted the experimental results for trivalent B1.8 binding. Data from curves with resonance levels that approach R_max_ would be close to those obtained for monovalent binding. In this case, as the concentration of trimerbody B1.8 increased, the calculated k_a_ diminished from 4×10^5^ M^−1^ s^−1^ (12.5 nM trimerbody) to 2×10^4^ M^−1^ s^−1^ (800 nM trimerbody). These changes are compatible with changes in the proportion of antibody that binds the ligand mono- or multivalently, since approaching surface saturation leads to a larger proportion of monovalent antibody binding. The apparent k_d_ had narrower range, from 6×10^−4^ (12.5 nM) to 9×10^−4^ (800 nM). Considering that at 12.5 nM, most of the trimerbody can bind bi- or multivalently, the functional affinity of the trimerbody calculated from this concentration could be K_D_ = 1.5×10^−9^ M or higher.

**Figure 2:**
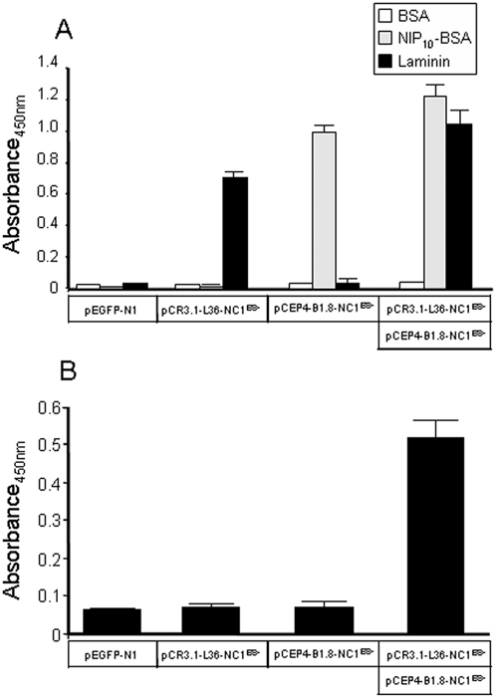
Binding activities of monospecific and bispecific trimerbody molecules. Supernatant from single-transfected [pEGFP-N1, pCR3.1-L36-NC1^ES-^, or pCEP4-B1.8-NC1^ES-^] 293T cells are compared with supernatant from double-transfected [pCR3.1-L36-NC1^ES-^ and pCEP4-B1.8-NC1^ES-^] 293T cells by direct ELISA (A), using plates coated with BSA, NIP-BSA conjugates (NIP_10_-BSA), and murine EHS laminin; and by sandwich-ELISA (B) using plates coated with laminin.

To further assess that at least two antigen-binding sites were located on the same trimerbody molecule, 293T cells were transfected with plasmid encoding L36 trimerbody, B1.8 trimerbody or cotransfected with both plasmids. Western blot analysis showed that the amount of trimerbody was higher in double-transfected than in single-transfected 293T cells (data not shown). Conditioned media from single-transfected 293T cells bound NIP_10_-BSA or laminin, whereas conditioned media from double-transfected 293T cells recognized both antigens (Fig. 2A). In sandwich ELISA, conditioned medium from double-transfected 293T was added to laminin-coated wells and, after washing, the laminin-bound trimerbodies were able to capture soluble NIP_10_-BSA (Fig. 2B). Trimerbodies in the supernatant from 293T cells single-transfected with plasmid encoding L36 trimerbody were shown to bind to laminin, but the bound trimerbody did not capture, in turn, NIP_10_-BSA.

### Stability *in vitro*

**Figure 3:**
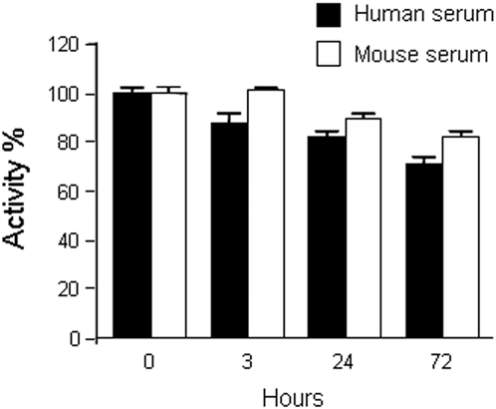
Serum stability of trimerbody molecules. Purified L36 trimerbody was incubated in human or mouse serum at 37°C, as explained in material and methods, and the reaction mixtures were analyzed by ELISA.

Testing the stability of engineered antibody fragments in serum is critical to determine their potential application *in vivo*. We therefore determined the functionality of L36 trimerbody after incubation in mouse and human serum at 37°C for prolonged periods of time. As shown in Fig. 3, L36 trimerbody retained 80–90% of its binding activity after 72 hours of incubation.

### Tumor Targeting

**Figure 4:**
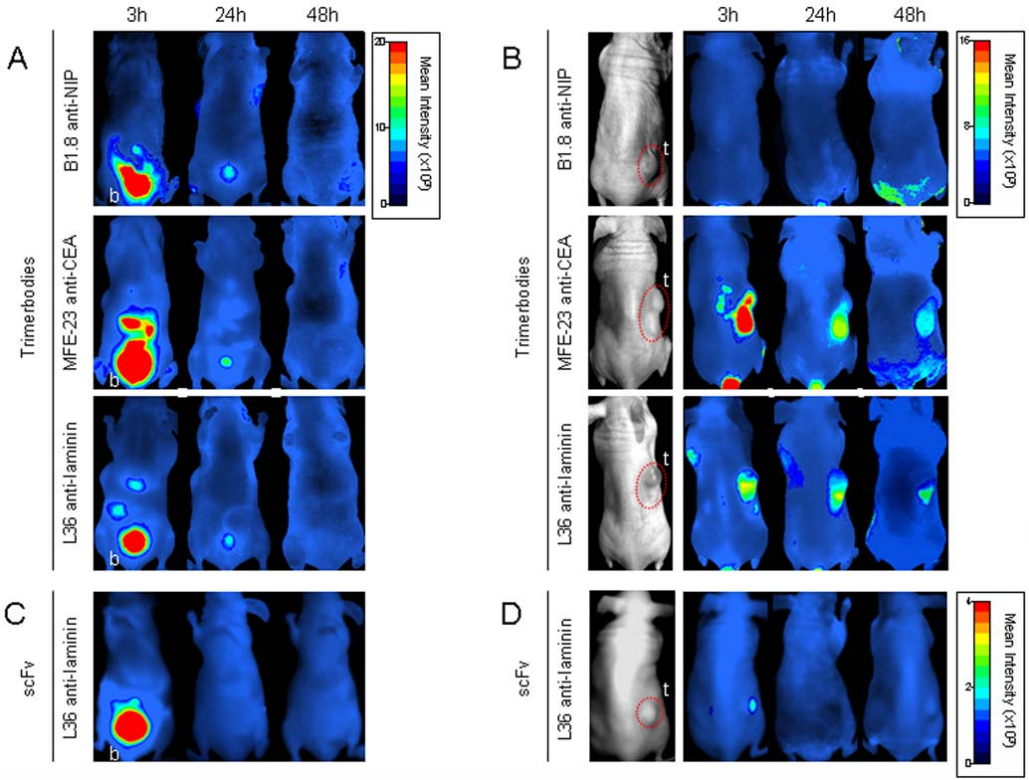
Targeting of fluorescently labeled antibodies to CEA-positive human tumor xenografts in nude mice. Near-infrared fluorescence imaging of nude mice bearing dorsal subcutaneous (s.c.) MKN45 human gastric-carcinoma tumors. Ventral (A, C) and dorsal (B, D) imaging was performed at 3, 24, and 48 hours after intravenous (i.v.) injection with Cy5-labeled trimerbodies (A, B), or Cy5-labeled L36 scFv (C, D). b: bladder, t: tumor.

To evaluate the tissue distribution and tumor targeting of anti-NIP, anti-CEA and anti-laminin trimerbodies we used an optical molecular imaging system, which allows a kinetic evaluation of tumor targeting and antibody clearance in the same animal imaged at different time points. Trimerbodies were labeled with the near-infrared fluorochrome Cy5 and injected in the tail vein of nude mice bearing MKN45 (stomach adenocarcinoma), HT-1080 (fibrosarcoma), or HeLa (cervical adenocarcinoma) human xenografts (n = 4/group). All trimerbodies showed a rapid renal clearance after i.v. injection, with peak signal intensity at 3 h and no detectable bladder signal at 48 h postinjection (Fig. 4A and C). Elimination of the L36-Cy5 scFv was even more rapid, and fluorescence was not detected in the bladder 24 hours after injection (Fig. 4C).

**Figure 5:**
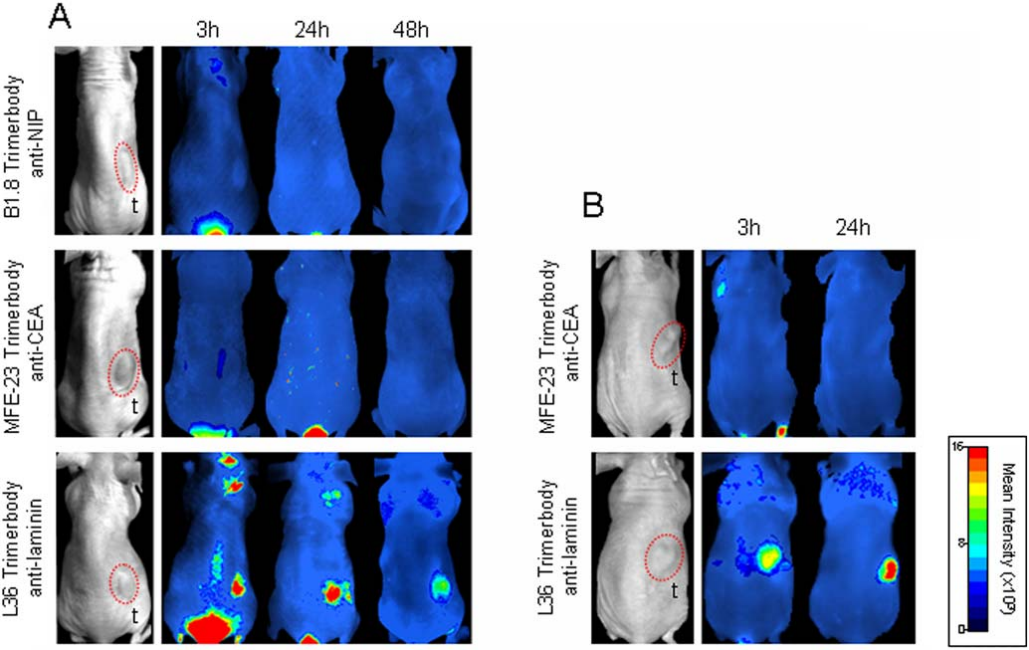
Targeting of fluorescently labeled trimerbodies to CEA negative human tumor xenografts in nude mice. Near-infrared fluorescence imaging of nude mice bearing s.c. HT1080 human fibrosarcoma tumors (A) or HeLa human cervical adenocarcinoma tumors (B). Imaging was performed at 3, 24, and 48 hours after i.v. injection with Cy5-labeled trimerbodies.

The control B1.8 trimerbody showed no detectable localization in any of the three tumor types studied (Fig. 4B and 5A). A strong and selective accumulation in CEA-positive tumors was observed in the case of the MFE-23 trimerbody. After i.v. injection of MFE23-Cy5 trimerbody, maximum tumor uptake was detected at 3 h, whereas the signal intensity decreased by 24 h and remained detectable for at least 48 h (Fig. 4B). The anti-laminin trimerbody showed localization in all the tumors studied, although the kinetics of signal onset differed from that observed with anti-CEA trimerbodies (Fig. 4B and 5). Maximum tumor uptake was at 24 h, which was lower than that of the MFE-23 trimerbody. The anti-laminin scFv also showed specific tumor uptake but at a much lower level that of the anti-laminin trimerbody (Fig. 4B and 4D).

## Discussion

Our previous studies showed the ability of collagen-derived sequences to promote antibody trimerization [15]. We reported that fusion of the N-terminal association subdomain of collagen XVIII NC1, responsible for the non-covalent trimerization of collagen XVIII alpha chains, to the C-terminus of a scFv antibody confers a trimeric state to the fused antibody (“trimerbody”). Using a scFv (L36) that recognizes an angiogenesis-associated laminin epitope [21] and inhibits tumor angiogenesis and growth [20], we demonstrated that locally produced trimeric L36 was more effective than its monomeric counterparts in blocking capillary morphogenesis *in vitro*, and in preventing tumor growth *in vivo* [15]. Recently, another group [32] has used a similar approach to drive antibody multimerization. They demonstrated that a short collagen-like peptide scaffold was able to promote trimerization of fused scFv fragments (“collabody”).

In this study, we present the most comprehensive characterization to date (both *in vitro* and *in vivo*) of the antibody-NC1 fusion, and we extend the concept by making analogous molecules with specificity for the hapten NIP and the human tumor-associated antigen CEA. All the trimerbodies were isolated in functional active form from conditioned medium of transfected HEK293 cells and easily purified using immobilized metal affinity chromatography. Purified trimerbodies are trimeric in solution, and exhibit excellent antigen binding capacity and stability. Trimerbodies are very efficient recognizing antigen when immobilized, or associated to the tumor cell surface. SPR analysis demonstrated that the trimerbody had a higher binding signal than the monomeric antibody and apparently slower dissociation, consistent with multivalent binding to the antigen. We calculated that the anti-NIP trimerbody has at least a 100-fold increase in apparent functional affinity for NIP-BSA conjugates compared to its monovalent counterpart. This result suggests that this affinity gain might be conferred by avidity effects of a second combining site in the trimerbody molecule. The presence of at least two functional binding sites in one single trimerbody molecule was further demonstrated in bispecific trimerbodies. Stable bifunctional anti-laminin x anti-NIP trimerbodies were easily produced by the coexpression of two different trimerbody constructs in human cells.

**Figure 6:**
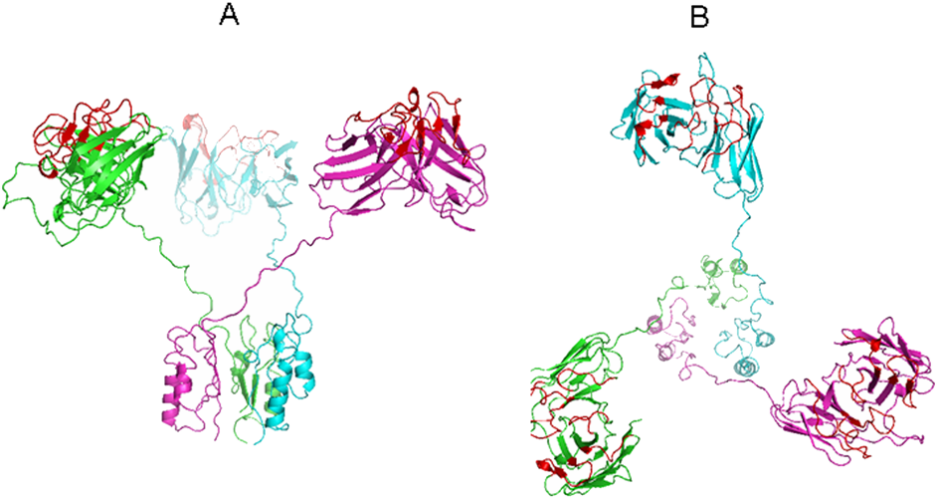
Structural model of the anti-laminin L36 trimerbody. Lateral (A) and superior view (B) of the molecular model of the anti-laminin L36 trimerbody. In this computer simulation, each monomer is colored differently (green, blue, and purple). The complementarity-determining regions (CDRs) are colored red.

The gain in affinity through avidity makes trimerbodies attractive for *in vivo* imaging as an alternative reagent to dimeric antibodies. It is tempting to speculate that trimerbodies will be preferred over dimeric antibodies (diabodies and minibodies), although this property may be dependent on the structure and density of the antigen recognized by the scFv modules. For full avidity in multivalent antibodies targeted to surface-bound molecules, the antigen binding sites must point out towards the same direction. If simultaneous multiple binding is not sterically possible, then apparent gains in functional affinity are likely to be small and due only to the effect of increased rebinding, which is dependent on diffusion rates and surface antigen concentration [33]. Analysis of the trimerbody model suggests a tripod-shaped structure with the scFv domains outward oriented (Fig. 6). Flexibility between antigen binding sites is another important aspect in the design of multivalent antibodies required to cross-link surface receptors on either the same or adjacent cells [2]. The twenty-one residue linker, with a maximal length of 79.8 Å if the conformation is fully extended [34], is very flexible allowing numerous binding geometries. When an antigen-antibody interaction takes place the possibility of establishing a second interaction depends on the valence, orientation and flexibility of the antigen-binding site. Multiple binding can effectively reduce the off rates thereby increasing the retention time of the antibody bound to the target antigen. In this respect, a major advantage of trimerbody over other trimeric formats (e.g. collabody) is the flexibility. The more compact/rigid collabody structure reduces accessibility of the scFv modules [15], that is a critical parameter for tumor targeting *in vivo*.

Thus, multimerization of scFv contructs has advantages for *in vivo* applications. Multivalent recombinant antibodies, such as diabodies and minibodies have shown promise as *in vivo* targeting agents [2]. Trimerbodies are intermediate-sized, multivalent molecules that exhibit high stability under physiological conditions. The potential of trimerbody molecules for *in vivo* targeting was assessed in nude mice bearing human tumor xenografts. Anti-CEA trimerbody localized rapidly and specifically to CEA-positive tumor xenografts. The tumor uptake reached a maximum at 3 hours postinjection and slowly washed out over time. Fluorescence was still detectable in the tumor at 48 hours after trimerbody injection. Importantly, the anti-laminin L36 trimerbody localized in all the tumors studied independently of tumor type. Maximum tumor uptake of anti-laminin trimerbodies was at 24 h following administration. Although the anti-laminin L36 scFv showed specific tumor accumulation, the tumor uptake was limited, probably due to the rapid clearance from the blood pool (with half-lives less than 15 min) and its monovalent nature (involving low retention times) [35]–[38].

According to our previous results, the L36 epitope is located in the middle part of the rod-like portion of the laminin long arm, in a highly flexible area, which corresponds to a protease-susceptible site [39]. We have postulated that this epitope is only exposed during basement membrane (BM) assembly [39], where polymerizing intact laminin acts as a scaffold for the recruitment of other BM components [40]. The restricted expression of this epitope, to situations associated with BM remodeling, would explain the slower tumor uptake of L36 trimerbodies compared with trimerbodies targeting tumor-associated antigens, and the reduced renal uptake of L36 anti-laminin trimerbodies compared with other anti-laminin antibodies in the literature [41].

Beyond its diagnostic applications, trimerbodies offer promising therapeutic opportunities on the basis of the selective delivery of bioactive molecules to the target tissue. Some of the immediate applications of trimerbodies specific for tumor-associated antigens (e.g. human epidermal growth factor receptor 2, prostate-specific antigen) or targeting tumor stroma (e.g., fibroblast activation protein) and neovasculature (e.g VEGFR-2/KDR or fibronectin extra-domain B) include the development of fusion proteins with angiogenic inhibitors [15], cytokines, enzymes, or truncated receptors, and conjugation with radionuclides [42].

## Supporting Information

Figure S1 Schematic structure of the scFv-NC1 gene (trimerbody).
(0.13 MB TIF)
