# Supplementary figures and images for: In Vivo Tumor Targeting and Imaging with Engineered Trivalent Antibody Fragments Containing Collagen-Derived Sequences
Source: PLoS One. 2009 Apr 29;4(4):e5381. doi: 10.1371/journal.pone.0005381 (PMC2670539; doi:10.1371/journal.pone.0005381)

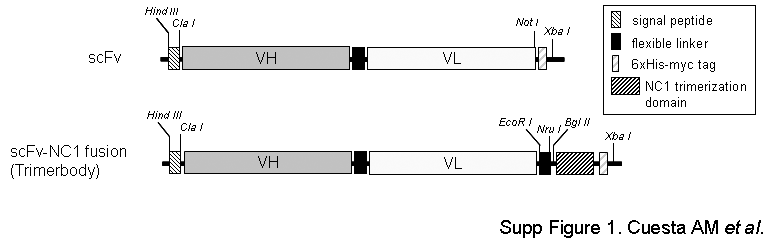

Supplement: Figure S1 — Schematic structure of the scFv-NC1 gene (trimerbody). (0.13 MB TIF) [file pone.0005381.s001.tif]
